# CD8+CD103+ tissue-resident memory T cells convey reduced protective immunity in cutaneous squamous cell carcinoma

**DOI:** 10.1136/jitc-2020-001807

**Published:** 2021-01-21

**Authors:** Chester Lai, George Coltart, Andrew Shapanis, Conor Healy, Ahmad Alabdulkareem, Sara Selvendran, Jeffrey Theaker, Matthew Sommerlad, Matthew Rose-Zerilli, Aymen Al-Shamkhani, Eugene Healy

**Affiliations:** 1 Dermatopharmacology, Faculty of Medicine, University of Southampton, Southampton, UK; 2 Dermatology, University Hospital Southampton NHS Foundation Trust, Southampton, UK; 3 Cancer Sciences, Faculty of Medicine, University of Southampton, Southampton, UK; 4 Histopathology, University Hospital Southampton NHS Foundation Trust, Southampton, UK; 5 Institute for Life Sciences, Faculty of Medicine, University of Southampton, Southampton, UK; 6 Centre for Cancer Immunology, University of Southampton, Southampton, UK

**Keywords:** skin neoplasms, T-Lymphocytes, lymphocytes, tumor-infiltrating, tumor microenvironment, CD8-positive T-lymphocytes

## Abstract

**Background:**

Tumor infiltrating lymphocytes play a key role in antitumor responses; however, while several memory T-cell subtypes have been reported in inflammatory and neoplastic conditions, the proportional representation of the different subsets of memory T cells and their functional significance in cancer is unclear. Keratinocyte skin cancer is one of the most common cancers globally, with cutaneous squamous cell cancer (cSCC) among the most frequent malignancies capable of metastasis.

**Methods:**

Memory T-cell subsets were delineated in human cSCCs and, for comparison, in non-lesional skin and blood using flow cytometry. Immunohistochemistry was conducted to quantify CD103+ cells in primary human cSCCs which had metastasized (P-M) and primary cSCCs which had not metastasized (P-NM). TIMER2.0 (timer.cistrome.org) was used to analyze TCGA cancer survival data based on *ITGAE* expression. Immunofluorescence microscopy was performed to determine frequencies of CD8+CD103+ cells in P-M and P-NM cSCCs.

**Results:**

Despite intertumoral heterogeneity, most cSCC T cells were CCR7−/CD45RA− effector/resident memory (TRM) lymphocytes, with naive, CD45RA+/CCR7− effector memory re-expressing CD45RA, CCR7+/L-selectin+ central memory and CCR7+/L-selectin− migratory memory lymphocytes accounting for smaller T-cell subsets. The cSCC CD8+ T-cell population contained a higher proportion of CD69+/CD103+ TRMs than that in non-lesional skin and blood. These cSCC CD69+/CD103+ TRMs exhibited increased IL-10 production, and higher CD39, CTLA-4 and PD-1 expression compared with CD103− TRMs in the tumor. CD103+ cells were more frequent in P-M than P-NM cSCCs. Analysis of TCGA data demonstrated that high expression of *ITGAE* (encoding CD103) was associated with reduced survival in primary cutaneous melanoma, breast carcinoma, renal cell carcinoma, kidney chromophobe cancer, adrenocortical carcinoma and lower grade glioma. Immunofluorescence microscopy showed that the majority of CD103 was present on CD8+ T cells and that CD8+CD103+ cells were significantly more frequent in P-M than P-NM cSCCs.

**Conclusion:**

These results highlight CD8+CD103+ TRMs as an important functional T-cell subset associated with poorer clinical outcome in this cancer.

## Introduction

Keratinocyte skin cancer is the most common type of cancer in the USA, with an annual incidence of approximately 5.4 million,^1^ and the yearly cost of treating this type of skin cancer in the USA has been estimated at $4.8 billion.^2^ In genetically susceptible individuals, including those with variant *MC1R* genotype and/or fair skin, exposure to ultraviolet radiation induces alterations in cancer driver genes within keratinocytes, which enable the development of skin cancers and precancerous skin lesions.^3–5^ Dysfunctional cutaneous immunity is also a well-known risk factor for keratinocyte skin cancer, especially cutaneous squamous cell carcinoma (cSCC). The immune system plays a fundamental role in suppressing carcinogenesis and subsequent metastasis, and there is an increasing understanding of the importance of infiltrating T cells in cancer, which can enable immune-mediated destruction of the tumor. Indeed, checkpoint inhibitors can provide an effective therapeutic strategy in various cancers, including cSCC,^6^ by enhancing durable memory T-cell antitumor immune responses. However, there is a need for further research into the roles of tissue-resident memory T cells (TRMs) in mediating protective or pathogenic adaptive immunity.^7 8^ Human skin is populated with approximately 20 billion resident T cells,^9^ which can provide protection against infection^10^ and melanoma^11 12^ and play a crucial role in the pathogenesis of inflammatory skin diseases such as psoriasis and vitiligo.^13^ Recently, multiple distinct memory T-cell subtypes with differing functional capacities have been identified in the skin.^14^ Although single-cell RNA sequencing of human cSCCs has demonstrated immunosuppresive Tregs and exhausted T cells within the tumor,^15^ it remains unclear what different memory T-cell subtypes infiltrate cSCCs and their functional relevance. An increased understanding of the composition of the cSCC T-cell infiltrate would provide greater insight into the immunopathogenesis of this common cancer and could lead to identification of potential novel therapeutic targets.

In this study, we performed phenotypic characterization of memory T cells in 80 freshly excised cSCCs (with matched blood±non-lesional skin (NS)) and 103 formalin-fixed paraffin-embedded cSCCs. We identify that CD8+CD103+ TRMs, which accumulate in cSCCs in higher frequencies than NS, upregulate expression of IL-10, CD39, CTLA-4 and PD-1, and that increased CD103+ and CD8+CD103+ cell frequencies are significantly associated with the development of metastases from primary cSCCs. These results indicate that CD8+CD103+ TRMs form an important dysfunctional T-cell subset that is associated with an adverse prognosis in cSCC.

## Materials and methods

### Flow cytometry

Fresh cSCC tumor tissue, NS and peripheral blood were obtained for flow cytometry from patients (n=72) in the Dermatology Department, University Hospital Southampton NHS Foundation Trust, Southampton, UK, and isolation of T cells from these samples was performed as described previously.^16^ Briefly, tumor and skin samples were disaggregated, treated with 1 mg/mL collagenase I (Sigma) and 10 µg/mL DNAse I (Sigma), passed through a 70µm cell strainer (BD) and centrifuged. The resulting pellet was then suspended and centrifuged over an Optiprep (Axis-Shield) density gradient and the buffy coat was extracted and washed with PBS. Peripheral blood mononuclear cells (PBMCs) were obtained by centrifuging peripheral blood layered on Lymphoprep (Axis-Shield) and the resulting buffy coat was collected and washed with PBS. Following incubation with PBS+1% BSA+10% FBS for 20 min, PBMCs and lymphocytes from tumor and NS were stained for 20 min in PBS+1% BSA+10% FBS with the following fluorophore-conjugate antibodies for cell surface markers and corresponding isotype controls: CD3 APC-Cy7 or PE-610 (Biolegend), CD4 FITC or APC-Cy7 or PerCP-Cy5.5 (Biolegend), CD8 PE-Cy7 (Biolegend), FOXP3 APC (ThermoFisher Scientific), CD45RA APC (Biolegend), CD45RO PerCP-Cy5.5 (Biolegend) or PE (BD Biosciences), CCR7 PE (Biolegend), CD27 APC-Fire-750 (Biolegend), CD28 APC (Biolegend), CD69 APC-Cy7 or PerCP-Cy5.5 (Biolegend), CD103 FITC (Biolegend), CD39 PE (Biolegend), PD-1 APC or PerCP-Cy5.5 (Biolegend), Tim3 PE (R&D Systems), LAG3 PE (R&D Systems), BTLA APC (Biolegend), CD160 PE (ThermoFisher Scientific), CD244 PE (ThermoFisher Scientific), TIGIT PE (Biolegend), CLA BV421 or FITC (BD Biosciences), CCR4 PerCP-Cy5.5 (Biolegend) or FITC (R&D Systems) and L-selectin BV421 or APC (Biolegend). Intracellular staining for CTLA-4 PE or BV421 (Biolegend) was done after staining of cell surface markers and subsequent fixation and permeabilization (Fixation and Permeabilization Kit, ThermoFisher Scientific). For detection of cytokines, cells were stimulated with PMA and ionomycin (Cell Stimulation Cocktail, ThermoFisher Scientific) in RPMI+10% FBS at 37°C for 5 hours in the presence of 3 µg/mL Brefeldin A (ThermoFisher Scientific) for the last 4 hours of culture. Cells were subsequently washed, fixed and permeabilized and stained with the following fluorophore-conjugated antibodies: IFNγ PE, BV421 or PerCP Cy5.5, TNFα FITC or PerCPCy5.5, IL-2 PE or BV421 and IL-10 PerCP Cy5.5 (all Biolegend). An aqua live dead viability stain was used (ThermoFisher Scientific). After staining, cells were washed, resuspended in PBS+1% BSA and analyzed with a BD FACSAria IIu flow cytometer. FlowJo software was used for data analysis.

### Immunofluorescence microscopy/confocal microscopy of frozen tissue sections

cSCC tissue samples (n=8 tumors) obtained from patients attending the Dermatology Department, University Hospital Southampton NHS Foundation Trust, Southampton, UK were snap frozen in liquid nitrogen, embedded in OCT (CellPath) and cut to 5–10 µm cryosections onto APES-coated slides. Sections were fixed in 4% paraformaldehyde, washed with PBS, blocked with PBS+1% BSA+10% FBS and incubated for 30 min with primary antibodies to the following markers: CD3 (rabbit polyclonal or mouse IgG1, both from Dako), CD4 (mouse IgG1, Abcam), CD8 (mouse IgG2a, ThermoFisher Scientific), CD45RO (mouse IgG2a, Novus Biologicals), CD103 (mouse IgG1, ThermoFisher Scientific), CD39 (rabbit polyclonal, Abcam), PD-1 (mouse IgG1, Abcam) and PD-L1 (rabbit polyclonal, Abcam). After washing off the primary antibodies, secondary antibodies were added; these included Alexa Fluor 488 or Alexa Fluor 555 goat antimouse IgG1a, Alexa Fluor 488 or Alexa Fluor 555 goat antirabbit IgG and Alexa Fluor 633 goat antimouse IgG2a (all from ThermoFisher Scientific). Sections were then counterstained with DAPI (Sigma), mounted with Mowiol (Harco), coverslipped and imaged using an Olympus Dotslide scanning fluorescence microscope or a Leica SP5 confocal microscope. Images were analyzed with Olympus VS-Desktop software for immunofluorescence microscopy and LAS AF for confocal microscopy.

### Immunostaining of formalin-fixed paraffin-embedded cSCCs

Formalin-fixed paraffin-embedded primary cSCCs which metastasized (n=47) and cSCCs which had not metastasized for at least 5 years following excision (n=56) were obtained from Histopathology, University Hospital Southampton NHS Foundation Trust. cSCCs were cut to 4 µm sections on APES-coated slides. After deparaffinization, rehydration and blocking of endogenous peroxidase, microwave antigen retrieval was performed using high pH target retrieval solution (Dako). Following this, slides were blocked with avidin (Vector), biotin (Vector) and then blocking solution containing 1% BSA and 10% FBS in DMEM was applied. A rabbit anti-CD103 primary antibody (Abcam) was applied to the slides overnight. After 3×5 min PBS washes, slides were incubated with a swine antirabbit biotinylated secondary antibody (Dako) for 30 min. Following three PBS washes, streptavidin-biotin-peroxidase complexes (Vector) were added for 30 min, then washed with PBS. For immunohistochemistry, DAB (Dako) was applied to the slides, which were then counterstained with Mayer’s Haematoxylin (Sigma), dehydrated and coverslipped. For CD8 and CD103 double immunofluorescence staining, after the rabbit anti-CD103 primary antibody and the swine antirabbit biotinylated secondary antibody steps, AlexaFluor 555-streptavidin (ThermoFisher Scientific) was applied and washed before incubation with mouse anti-CD8 (Abcam) for 1 hour. After 3 PBS washes, an AlexaFluor 488 goat antimouse secondary antibody (ThermoFisher Scientific) was applied. Sections were counterstained with DAPI, mounted in Mowiol and coverslipped. Slides were imaged using an Olympus Dotslide scanning microscope. Olympus VS-Desktop and ImageJ were used for image analysis. The number of stained cells was quantified in five representative images per tumor at 40 x magnification.

### Statistical/data analysis

GraphPad Prism was used for data analyzes and statistical calculations. Paired or unpaired analysis of variance with Tukey’s test for multiple comparisons was performed for analysis of flow cytometric quantification of normally distributed data, and Mann-Whitney test for comparing CD103+ cell numbers determined by immunohistochemistry between the primary metastatic and non-metastatic groups. Log rank test was used for Kaplan-Meier analysis. TIMER2.0 (timer.cistrome.org) Gene Outcome module was employed for analysis of TCGA cancer survival data based on *ITGAE* expression using a Cox proportional hazards model.^17^ Maximally selected rank statistics provided in survminer R package were used to determine the optimal cutpoints between groups in survival analyzes. The RNA-Seq by Expectation Maximization package scaled estimate output provided by Firehose was multiplied by 10^6^ to calculate the transcripts per million, which was then used to plot survival curves.

## Results

### Most cSCC T cells have a TEM phenotype

**Figure 1:**
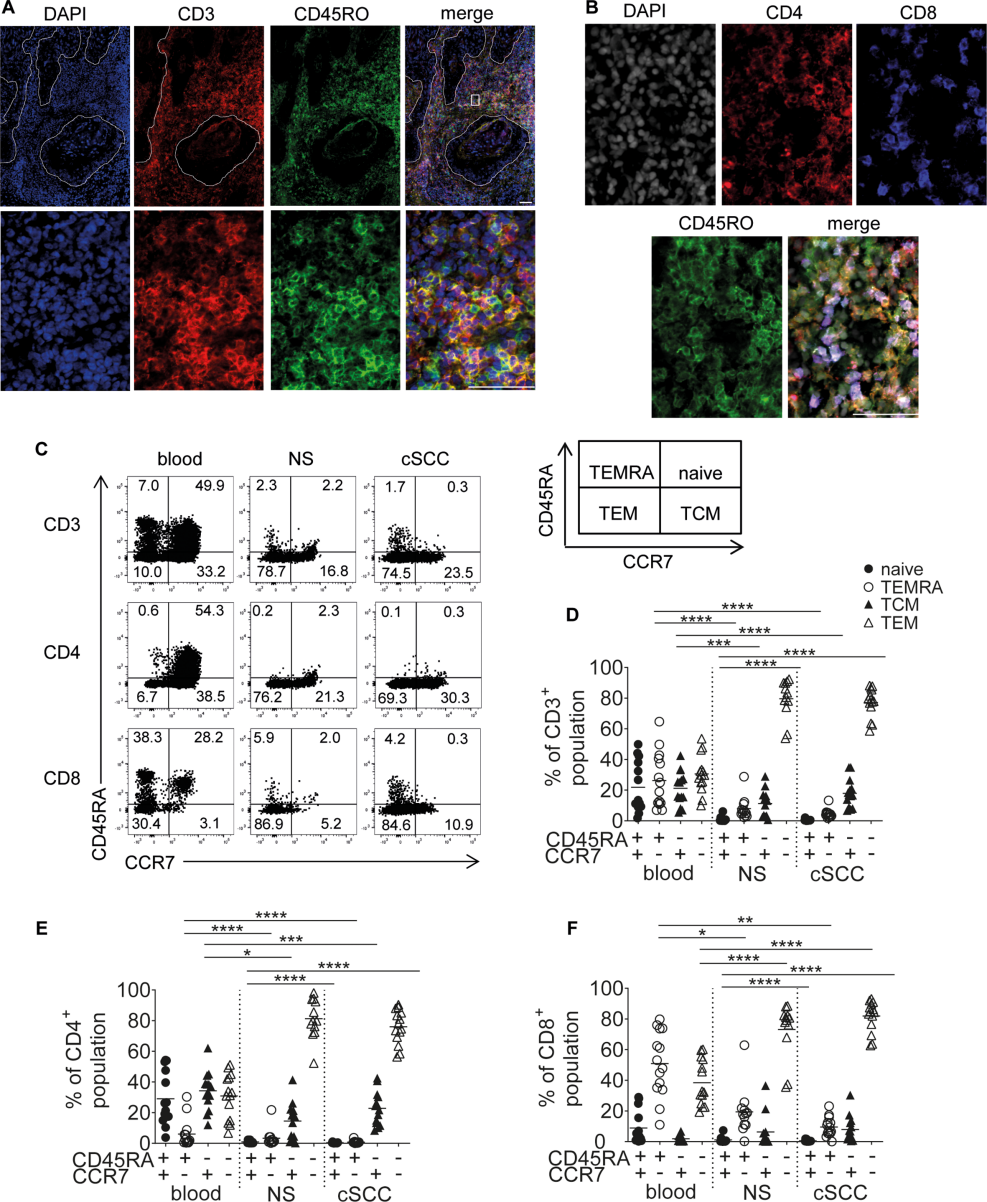
Most T cells in cSCC are CCR7−CD45RA− TEMs. (A) Representative immunofluorescence microscopy images of cSCC stained for DAPI, CD3 and CD45RO, top row—low magnification, bottom row—high magnification. Dashed lines indicate tumor island borders. (B) Representative immunofluorescence microscopy images of cSCC stained for DAPI, CD4, CD8 and CD45RO. (A and B), Scale bars=50 µm. (C) Representative FACS plots from blood, normal skin (NS) and cSCC from the same patient showing expression of CCR7 (X-axis) and CD45RA (Y-axis) in CD3+, CD4+ and CD8+ gated populations. A schematic diagram is shown on the right, defining the naive, TEMRA, TCM and TEM by CCR7 and CD45RA expression as depicted. (D–F) Graphs showing percentages of CD3+ (D), CD4+ (E) and CD8+ (F) T cells that are naive, TEMRA, TCM and TEM from blood, NS and cSCC (n=14 tumors). Horizontal bars=means, *p<0.05, **p<0.01, ***p<0.001, ****p<0.0001. cSCC, cutaneous squamous cell carcinoma; TCM, T-cell central memory; TEM, T-cell effector memory; TEMRA, T-cell effector memory re-expressing CD45RA.

Immunofluorescence staining to identify memory T cells in cSCC showed CD45RO colocalizing with CD3+ T cells, as well as with CD4+ and CD8+ cells, and that these CD45RO+ memory T cells were predominantly located in the stromal areas adjacent to tumor nests (figure 1A, B). CCR7 and CD45RA staining was used to distinguish four subpopulations of T cells:^18^ CCR7+CD45RA+ were considered naïve T cells, CCR7−CD45RA+ were effector memory re-expressing CD45RA (TEMRA), CCR7+CD45RA− were central memory (TCM) and CCR7−CD45RA− were effector memory (TEM, figure 1C–F). Most CD3+, CD4+ and CD8+ T cells in cSCC and NS were CCR7−CD45RA− TEMs (mean 77.2% of cSCC CD3+ population, 79.6% of NS CD3+ population, 76.0% of cSCC CD4+ population, 81.3% of NS CD4+ population, 81.9% of cSCC CD8+ population, 73.1% of NS CD8+ population, n=14 tumors). While cSCC and NS contained similar frequencies of TEMs, TCMs (mean 17.9% and 11.3% of CD3+ population, respectively), naïve T cells (mean 0.63% and 1.2% of CD3+ population, respectively) and TEMRAs (mean 4.5% and 8.0% of CD3+ population, respectively), there were significantly more TEMs and fewer naïve T cells and TEMRAs in cSCC and normal skin than peripheral blood (p<0.001 for all comparisons, figure 1D–F).

There was significantly higher expression of the skin-homing marker CLA on T-cell populations in cSCC and NS compared with blood (mean 52.7% and 61.4% vs 16.6% of CD3+ populations, respectively, p<0.0001 for both comparisons, n=14 tumors), but lower CLA+ frequencies were observed on CD8+ T cells from cSCC compared with NS (mean 42.7% vs 53.2% of CD8+ population, respectively, p=0.0454 (online supplemental figure 1A, B). CCR4, another skin addressing, was significantly less frequently expressed in T cells from cSCC and blood than those from NS (mean 25.0% and 18.3% vs 46.5% of CD3+ populations, respectively, n=17 tumors, p=0.0004 and p=0.0074, respectively (online supplemental figure 1B). CCR7 and L-selectin were used to identify CCR7+ L-selectin+ TCMs and CCR7+L-selectin− migratory memory (TMM) T cells, which have been shown to have the ability to recirculate between skin and blood^14^ (online supplemental figure 2A, B). There were significantly fewer CD3+ T cells that were CCR7+L-selectin+ TCMs in cSCC compared with blood (mean 10.3% vs 28.6%, respectively, n=10 tumors, p=0.0437), whereas the proportion of TMMs did not differ significantly between cSCC, normal skin and blood (means 10.7%, 5.7% and 14.7% of the CD3+ populations, respectively).

10.1136/jitc-2020-001807.supp1 Supplementary data

10.1136/jitc-2020-001807.supp2 Supplementary data

### Characterisation of CD27 and CD28 by T cells in blood, non-lesional skin and cSCC

**Figure 2:**
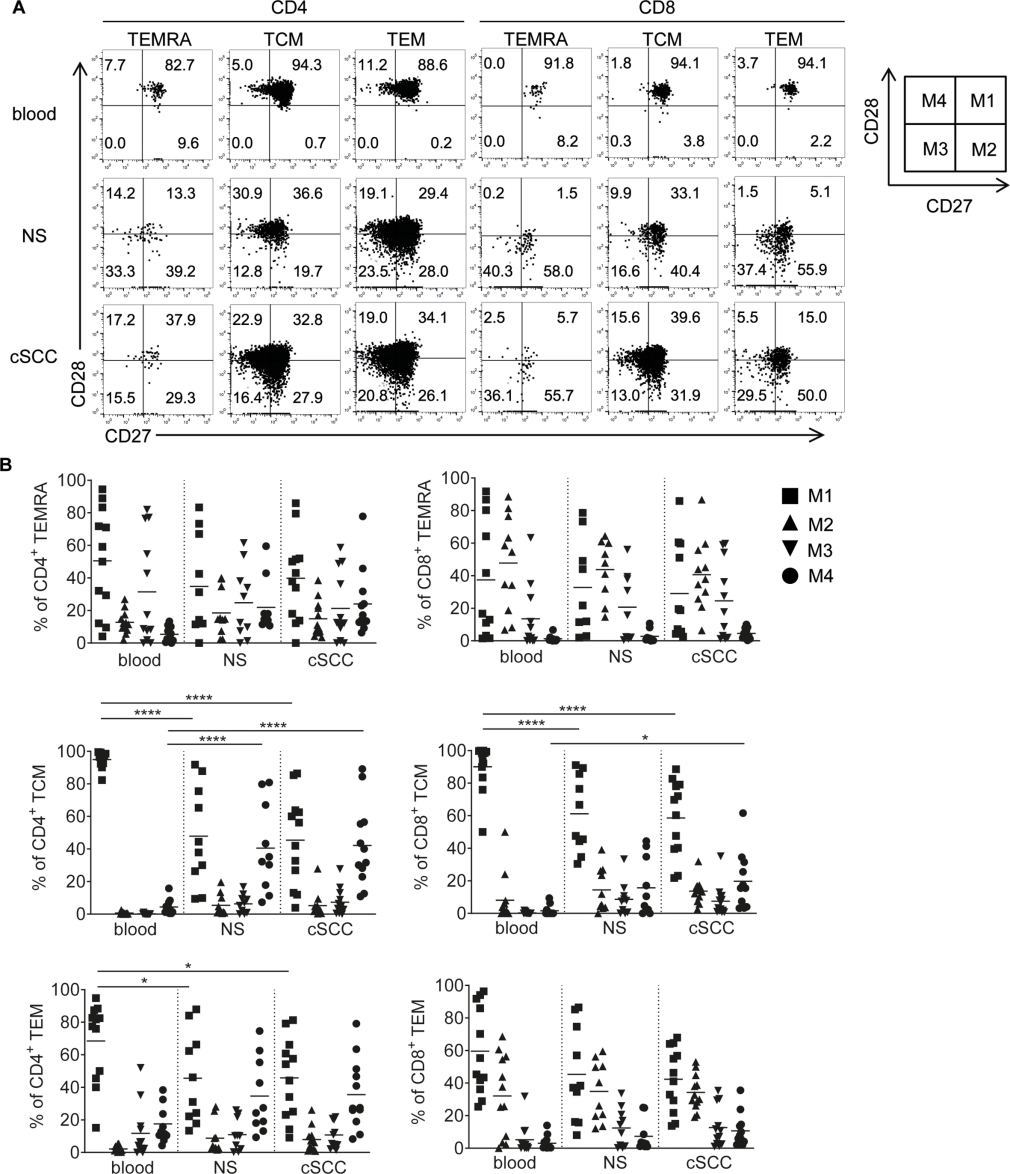
Expression of CD27 and CD28 by different memory T-cell subsets. (A) Representative FACS plots from blood, normal skin (NS) and cSCC from the same patient showing expression of CD27 (X-axis) and CD28 (Y-axis) in TEMRAs, TCMs and TEMs within the CD4 and CD8 T-cell populations. A schematic diagram is shown on the right, defining the M1 (CD27+CD28+), M2 (CD27+CD28−), M3 (CD27−CD28−) and M4 (CD27−CD28+) populations as depicted (with M1, M2, M3, M4 also known as EM1, EM2, EM3 and EM4 in the TEM population). (B) Graphs showing percentages of CD4+ TEMRAs (upper left), CD4+ TCMs (middle left), CD4+ TEMs (lower left) and CD8+ TEMRAs (upper right), CD8+ TCMs (middle right), CD8+ TEMs (lower right) from blood, NS and cSCC (n=14 tumors) which are M1, M2, M3 and M4. Horizontal bars=means, *p<0.05, ****p<0.0001. cSCC, cutaneous squamous cell carcinoma; TCM, T-cell central memory; TEM, T-cell effector memory; TEMRA, T-cell effector memory re-expressing CD45RA.

It has been shown that TEMs can be heterogenous and subdivided into four further subtypes based on CD27 and CD28 expression: EM1 (CD27+CD28+), EM2 (CD27+CD28−), EM3 (CD27−CD28−) and EM4 (CD27−CD28+).^19^ While EM1 and EM4 cells display characteristics akin to TCM, EM2 and EM3 cells resemble more differentiated effector T cells with increased cytolytic activity.^19^ Assessment of naïve (online supplemental figure 3), TEMRA, TCM and TEM in blood, NS and cSCC demonstrated mostly similar EM1, EM2, EM3 and EM4 profiles in the TEM populations in NS and cSCC (figure 2A and B; n=10 tumors). Extending the terminology to TEMRA and TCM so that in these cell types, CD27+ CD28+ cells were termed as M1, CD27+CD28− as M2, CD27−CD28− as M3 and CD27−CD28+ as M4, there were no significant differences between cSCC and NS in the frequencies of M1, M2, M3 and M4 cell populations. However, there were significant differences in CD27 and CD28 expression between cSCC and blood. For example, compared with peripheral blood, cSCCs were characterized by lower M1 fractions within the CD4 TCM (p<0.0001), CD4 TEM (p=0.0137) and CD8 TCM populations (p<0.0001), and higher M4 proportions in the CD4 TCM (p<0.0001) and CD8 TCM populations (p=0.0193). Among the CD4 naïve T-cell populations, there were significantly fewer CD27+CD28+ cells in cSCC and NS than blood (p<0.0001 for both comparisons), and more CD27−CD28+ cells in NS than blood (p=0.0043, online supplemental figure 3B).

10.1136/jitc-2020-001807.supp3 Supplementary data

### CD8+CD103+ TRMs are more frequent in cSCC than in blood and non-lesional skin

**Figure 3:**
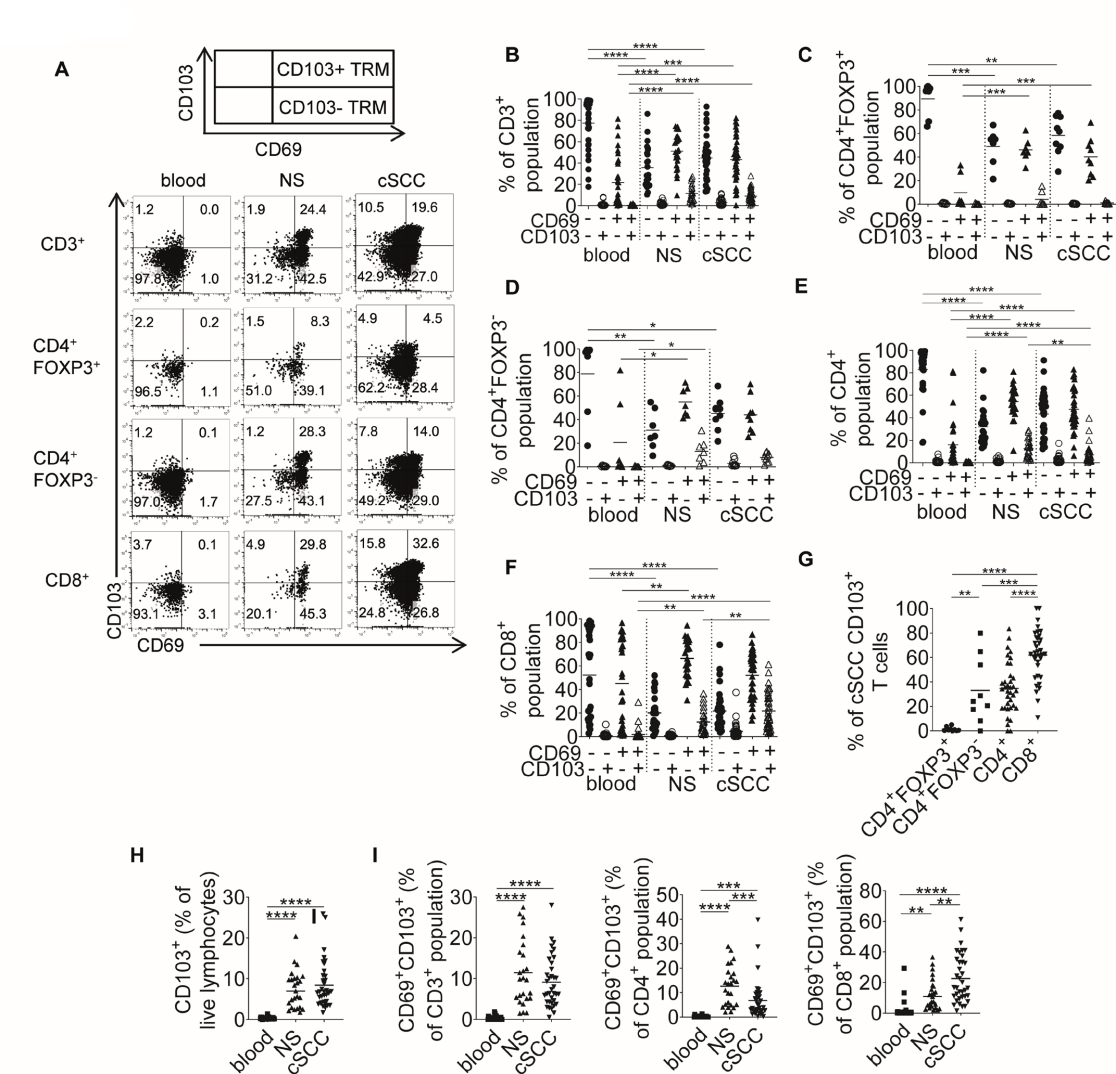
CD8+CD103+ TRMs are more frequent in cSCC than in blood and normal skin (NS). (A) Representative FACS plots from blood, NS and cSCC from the same patient showing expression of CD69 (X-axis) and CD103 (Y-axis) in CD3+, CD4+FOXP3+, CD4+ FOXP3− and CD8+ gated populations. (B–F) Graphs showing frequencies of cells based on CD69 and CD103 expression as percentages of (B) CD3+, (C) CD4+ FOXP3+, (D) CD4+ FOXP3−, (E) total CD4+ and (F) CD8+ populations. (G) Graph showing percentages of CD103+ T cells that are CD4+ FOXP3+, CD4+ FOXP3−, CD4+ and CD8+. (H) Percentages of the live lymphocyte population expressing CD103 in blood, NS and cSCC. (I) Percentages of the CD3+ (left), CD4+ (center) and CD8+ (right) T-cell populations from blood, normal skin and cSCC that are CD69+CD103+ TRMs. n=36 tumors for all graphs except (C) and (D), where n=9 tumors. Horizontal bars=means, *p<0.05, **p<0.01, ***p<0.001, ****p<0.0001. cSCC, cutaneous squamous cell carcinoma; TRMs, resident memory T cells.

TRMs in skin express CD69 and a functionally distinct subset of these TRMs also express CD103.^14^ In cSCC, we found that CD103 was mostly present on CD69+ TRMs (figure 3A–B). CD69−CD103− T cells accounted for significantly lower proportions of the CD3+ population in cSCC and NS compared with blood (mean 44.2% and 35.7% vs 77.4%, respectively: p<0.0001 for both comparisons, n=36 tumors, figure 3B). CD69+CD103− TRMs formed a greater proportion of the CD3+ population in cSCC and NS than blood (mean 43.4% and 51.2% vs 21.7%, respectively, cSCC vs blood: p=0.0002, NS vs blood: p<0.0001). The percentage of CD3+ T cells that were CD69+CD103+ TRMs was also higher in cSCCs and NS than blood (mean 9.1% and 11.4% vs 0.3%, respectively, p<0.0001, n=36 tumors, figure 3B). CD69 and CD103 expression was also examined on CD4+FOXP3+ regulatory T cells (Tregs) and CD4+FOXP3− T cells in a subset of cSCCs (n=9 tumors, figure 3C and D). Within the cSCC CD4+FOXP3+ Treg population, most were CD69−CD103− (mean 58.4%) or CD69+CD103− (mean 40.1%) TRMs, while CD69+CD103+ TRMs were infrequent (mean 1.1%, figure 3C). While greater numbers of CD69+CD103+ TRMs were present in the CD4+FOXP3− population (mean 7.9%, figure 3D), and in the total CD4+ population (mean 7.0%, figure 3E) than in the Treg group, CD69+CD103+ TRMs were present in greater amounts in the CD8+ population (mean 24.1%, figure 3F). Significantly higher percentages of tumor-infiltrating CD103+ T cells were CD8+ (mean 62.0%, figure 3G) than CD4+ (mean 34.8%, p<0.0001), CD4+FOXP3+ (mean 1.4%, p<0.0001) and CD4+FOXP3− (33.0%, p=0.0009). Although CD103+ TRMs accounted for similar percentages of the total lymphocyte and CD3+ T-cell populations in cSCC and NS, the percentage of CD4 T cells that were CD103+ TRMs was lower in cSCC than NS (p=0.0007, figure 3E, H, I), whereas, by contrast, cSCCs contained higher percentages of CD8 T cells that were CD103+ TRMs than NS (p=0.0039, figure 3F and I).

**Figure 4:**
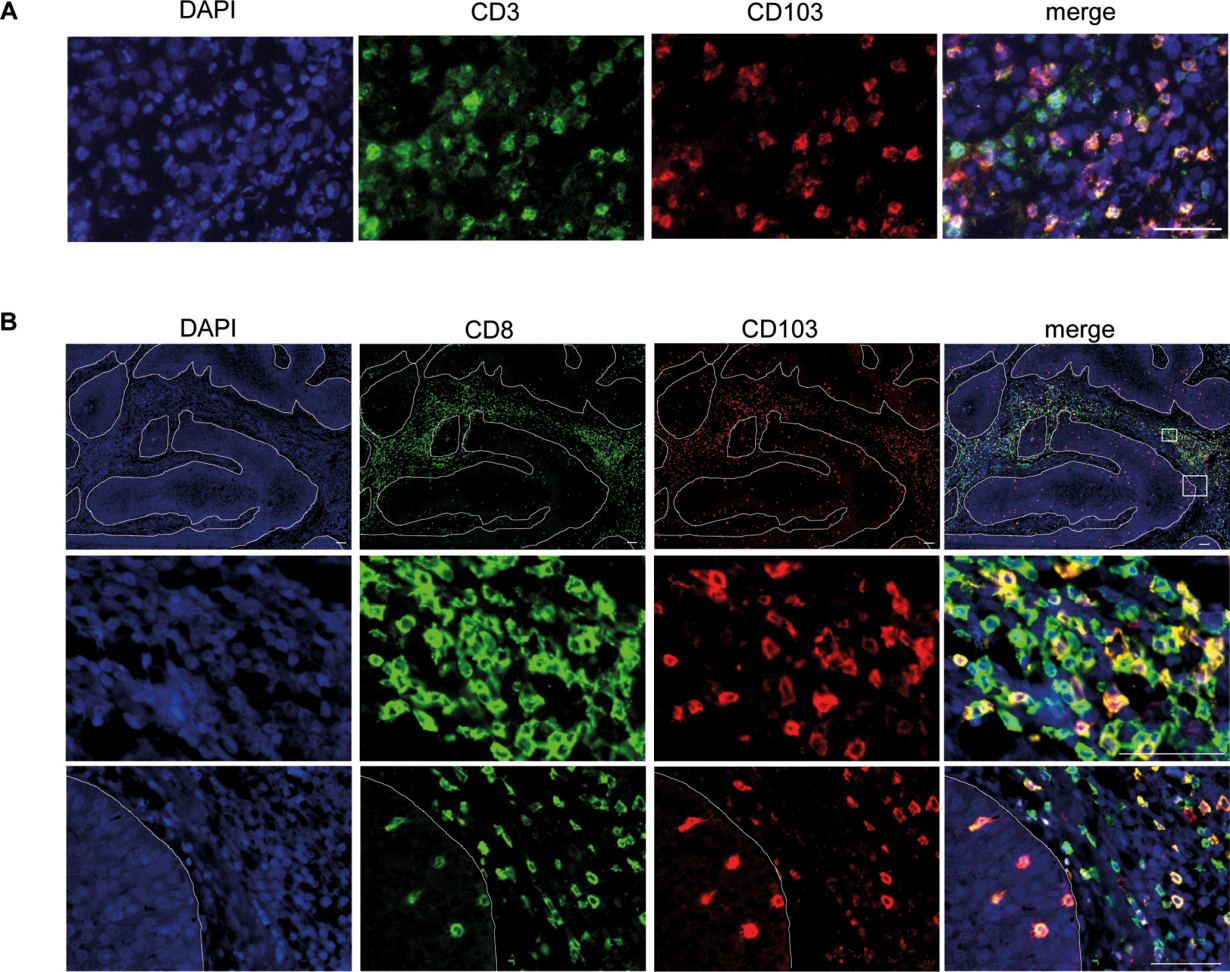
CD103+ TRMs in cSCC are situated predominantly in peritumoral areas. (A and B) Representative immunofluorescence microscopy images of cSCC showing (A) CD3 and CD103 expression and (B) CD8 and CD103 expression. In (B), the top row shows low magnification view, the middle row shows a high magnification view of a peritumoral region and the bottom row shows a high magnification view of a border between the tumor and peritumoral region. Dashed lines indicate tumor island borders. Scale bars=50 µm. cSCC, cutaneous squamous cell carcinoma; TRMs, resident memory T cells.

Immunofluorescence microscopy confirmed that the vast majority of CD103 expressing cells in cSCC coexpressed CD3 (figure 4A and online supplemental figure 4A) and many cSCC CD103+ cells also coexpressed CD8 (figure 4B and online supplemental figure 4B). These CD103+ TRMs were predominantly located in the peritumoral stromal areas, although there were smaller frequencies that were present in the tumor nests, where the vast majority of CD8 T cells expressed CD103 (figure 4B).

10.1136/jitc-2020-001807.supp4 Supplementary data

### cSCC CD8+CD103+ TRMs upregulate IL-10 production and expression of CD39, CTLA-4 and PD-1

**Figure 5:**
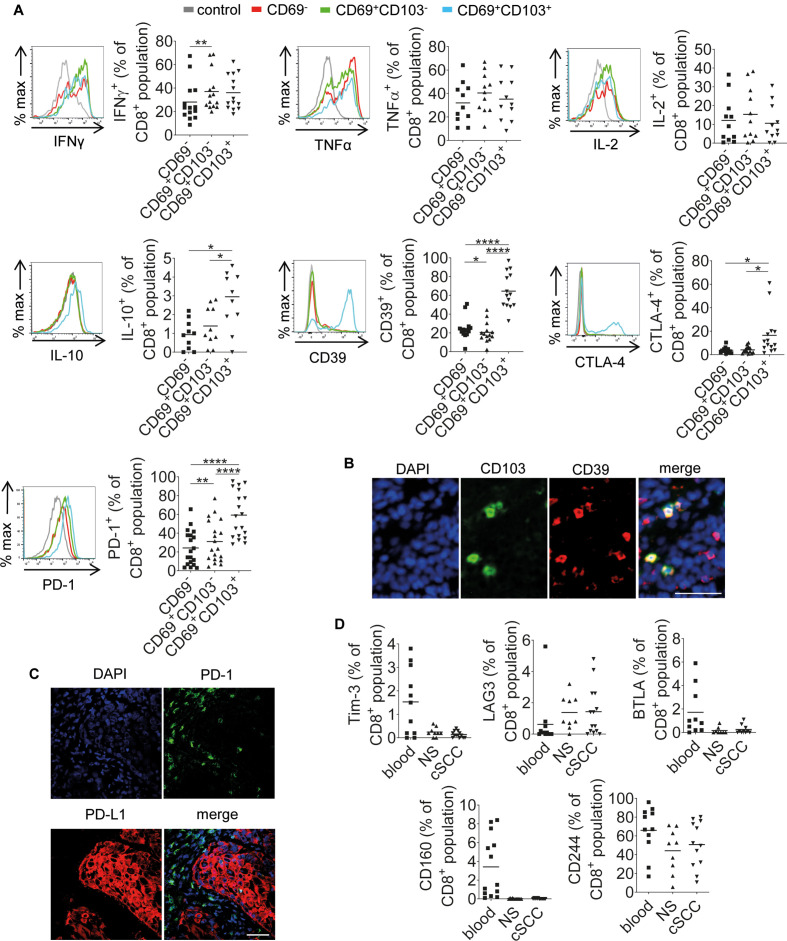
CD8+CD69+CD103+ TRMs in cSCC are associated with increased expression of IL-10, CD39, CTLA-4 and PD-1. (A) Representative FACS histograms and accompanying graphs showing expression of IFNγ (n=14 tumors), TNFα (n=11 tumors), IL-2 (n=11 tumors), IL-10 (n=10 tumors), CD39 (n=14 tumors), CTLA-4 (n=14 tumors) and PD-1 (n=11 tumors) by the tumor-infiltrating CD8+CD69−, CD8+CD69+CD103− and CD8+CD69+CD103+ T-cell populations. Expression of cytokines was determined following stimulation in vitro with PMA and ionomycin for 5 hours. Horizontal bars=means, *p<0.05, **p<0.01, ****p<0.0001. (B) Representative immunofluorescence microscopy images of cSCC stained for CD103 and CD39. (C) Representative confocal microscopy images of cSCC showing PD-1 and PD-L1 expression. (D) Percentages of CD8+ T cells from blood, normal skin (NS) and cSCC expressing Tim3 (n=13 tumors), LAG3 (n=14 tumors), BTLA (n=11 tumors), CD160 (n=13 tumors) and CD244 (n=12 tumors). (B and C) Scale bars=50 µm. cSCC, cutaneous squamous cell carcinoma; TRMs, resident memory T cells.

Lymphocytes isolated from cSCCs were stimulated with PMA and ionomycin and intracellular flow cytometry was performed to investigate the capacity of cSCC TRMs to produce IFNγ, TNFα, IL-2 and IL-10 (figure 5A). In the cSCC CD8 T-cell population, CD103+ TRMs produced similar amounts of IFNγ, TNFα and IL-2 compared with CD69− T cells and CD103− TRMs (figure 5A). However, IL-10 was expressed by significantly more tumor-infiltrating CD8+CD103+ TRMs than CD69− T cells (p=0.0239, n=10 tumors) and CD103− TRMs (p=0.0423, figure 5A). While IL-10 production was detected in a relatively small proportion of the CD8+CD103+ cells, it suggested a possible upregulation of an immunosuppressive phenotype by tumor-infiltrating CD103+ TRM, therefore expression of the inhibitory markers CD39, CTLA-4 and PD-1 was investigated. CD39, CTLA-4 and PD-1 was found to be present on significantly higher proportions of CD8+CD103+ TRMs than CD69− T cells and CD103− TRMs (mean CD39+ cells = 64.5% vs 24.7% and 20.6%, respectively, p<0.0001 for both comparisons, n=14 tumors; mean CTLA-4+ cells = 16.5% vs 3.8% and 4.1%, respectively, n=14 tumors, p<0.05 for both comparisons; mean PD-1+ cells = 59.2% vs 24.2% and 31.1%, respectively, n=19 tumors, p<0.0001 for both comparisons, figure 5A). Analysis of the tumor-infiltrating CD4+ T-cell population showed that CD4+CD103+ TRMs expressed IFNγ in higher frequencies than CD69− T cells, and TNFα in greater percentages than CD69− T cells and CD103− TRMs, but there were no significant differences between the CD4+CD103+ TRMs and the other T-cell subsets in the expression of IL-2 (online supplemental figure 5A-C). Although more of the CD4+CD103+ TRMs expressed IL-10, this did not reach statistical significance (n=10 tumors), but there was a significantly higher proportion of CD4+CD103+ TRMs than CD69− T cells and CD103− TRMs expressing CD39 (online supplemental figure 5DE). Additionally, PD-1 was present on larger proportions of CD4+CD103+ TRMs and CD4+CD103− TRMs than CD4+CD69− T cells (online supplemental figure 5F). However, CTLA-4 expression did not differ between the three CD4+ cell groups (online supplemental figure 5G). These results suggest that CD103+ TRMs, which are predominantly within the cSCC CD8 T-cell population, display a regulatory phenotype. Consistent with this, immunofluorescence microscopy confirmed CD103+ immune cells in cSCC also expressed CD39 (n=5 tumors, figure 5B and online supplemental figure 6).

10.1136/jitc-2020-001807.supp5 Supplementary data

10.1136/jitc-2020-001807.supp6 Supplementary data

Confocal microscopy of cSCCs demonstrated the exhaustion marker PD-1 on immune cells and its ligand PD-L1 was expressed by tumor cells (n=5 tumors, figure 5C). We also investigated whether other exhaustion markers were present on cSCC T cells. Tim-3, LAG3, BTLA and CD160 were expressed by a mean of <2% of tumor-infiltrating CD8 T cells, and while CD244 (2B4) was present on higher numbers of CD8 T cells in cSCC, the frequencies of CD244+CD8 T cells did not significantly differ between cSCC, NS and blood (figure 5D). Analysis of the tumor-infiltrating CD4 T-cell population also showed low expression of Tim-3, LAG3, BTLA and CD160 (mean<1%) and CD244 was found on 4.6% of tumor-infiltrating CD4 T cells, with no significant differences in expression identified between blood, NS and cSCC (online supplemental figure 7).

10.1136/jitc-2020-001807.supp7 Supplementary data

### Increased CD8+CD103+ cell frequencies are associated with metastasis

**Figure 6:**
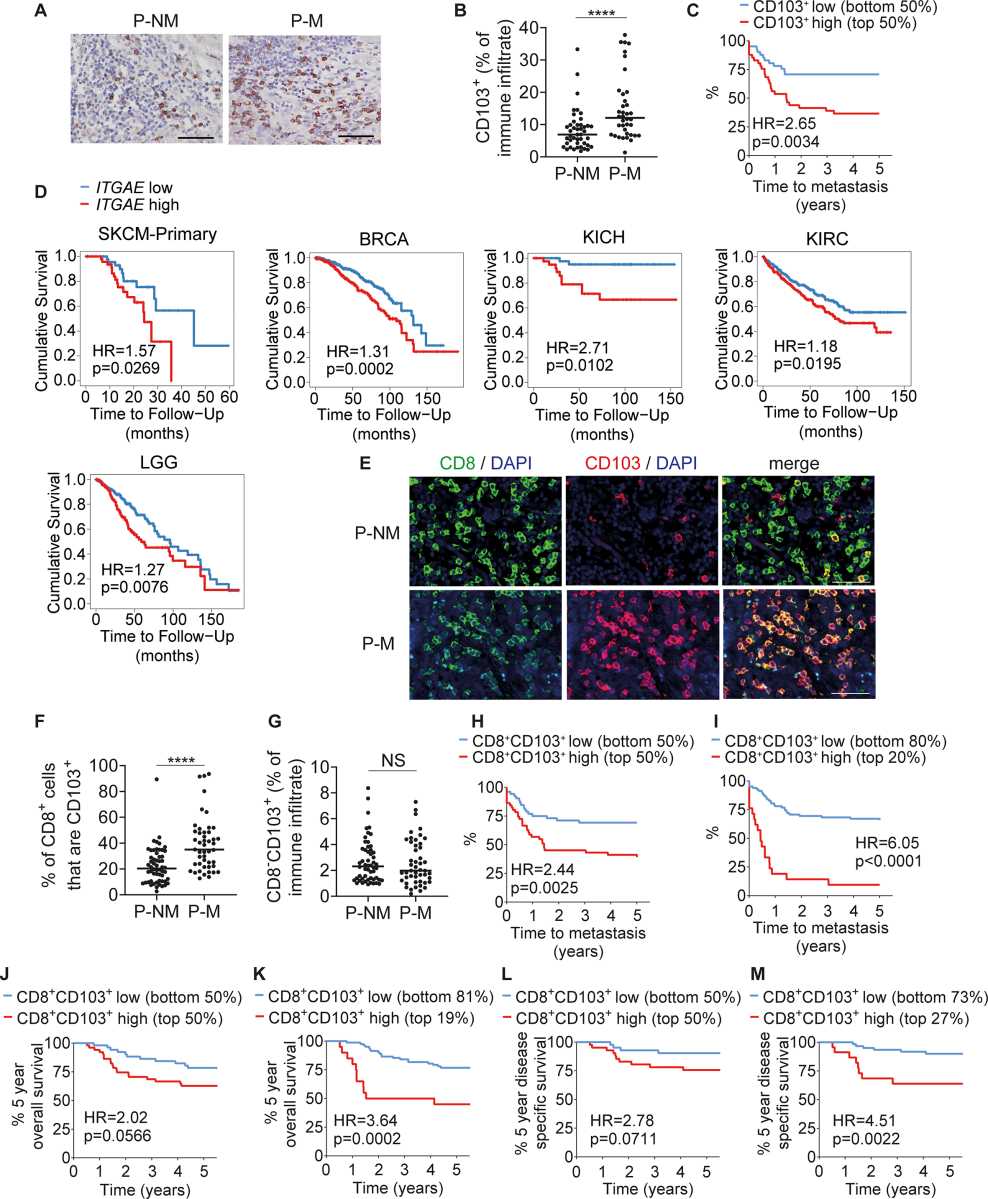
CD103+ TRMs in primary cSCCs are associated with development of metastasis. (A) Representative immunohistochemistry images of cSCC stained for CD103. (B) Percentages of tumor-infiltrating immune cells that are CD103+ in primary cSCCs that did not metastasize (P-NM, n=44) and primary cSCCs that metastasized (P-M, n=38). (C) Kaplan-Meier plot showing days to metastasis for the cSCCs in (B) stratified by low (<9.25% of immune infiltrate, n=41) and high (≥9.25% of immune infiltrate, n=41) expression of CD103. (D) Survival analyzes by *ITGAE* expression based on TCGA data in primary cutaneous melanoma (SKCM-Primary, n=103), breast carcinoma (BRCA, n=1100), kidney chromophobe cancer (KICH, n=66), kidney renal clear cell carcinoma (KIRC, n=533) and lower grade glioma (LGG, n=516). (E) Representative immunofluorescence microscopy images of cSCCs stained for CD8 and CD103. (F) Percentages of CD8+ cells that are CD103+ in P-NM (n=56) and P-M (n=47) cSCCs. (G) Percentages of immune cells that are CD8−CD103+ in P-NM and P-M cSCCs. (H, I) Time to metastasis for the cSCCs in (F) split into low and high CD103+ cell frequencies as a percentage of the CD8+ cell population divided at (H) the median (low ≤26.04% of CD8+ population, n=52; high >26.04% of CD8+ population, n=51) and (I) the most informative cutpoint based on maximally selected rank statistics (low <41.7% of CD8+ population, n=82; high >41.7% of CD8+ population, n=21). (J, K) 5-year overall survival data for cSCCs split into low and high CD8+CD103+ cell frequencies divided at (J) the median and (K) the optimal cutpoint based on maximally selected rank statistics (low <42.2% of CD8+ population, n=83; high >42.2% of CD8+ population, n=20). (L, M) Disease-specific survival data for cSCCs split into low and high CD8+CD103+ cell frequencies divided at (L) the median (low ≤24.2% of CD8+ population, n=43; high >24.2% of CD8+ population, n=42) and (M) the optimal cutpoint based on maximally selected rank statistics (low <34.9% of CD8+ population, n=62; high >34.9% of CD8+ population, n=23). In (L, M) cases where the exact cause of death was not known were excluded. In (A, E) scale bars=50 µm. In (B, F, G) horizontal bars=medians. ****p<0.0001; NS, not significant. cSCC, cutaneous squamous cell carcinoma.

To assess the association between CD103 expression in cSCC and clinical outcome, immunohistochemistry was performed on formalin-fixed paraffin-embedded sections of surgically excised primary cSCCs which subsequently metastasized (P-M, n=38) and surgically excised primary cSCCs which had not metastasized at the time of at least 5 years of patient follow-up (P-NM, n=44) in the dermatology/skin cancer clinics in our hospital. CD103 was expressed on immune cells infiltrating the cSCC stroma (figure 6A), and P-M cSCCs contained significantly increased percentages of CD103+ cells in the immune infiltrate compared with P-NM cSCCs (median 12.1% vs 6.9%, respectively, p<0.0001, figure 6B). cSCCs were then characterized into two groups based on CD103 expression—CD103 low (below median expression; CD103 expressed by <9.24% of immune infiltrate, n=41 tumors) and CD103 high (above median expression; CD103 expressed by ≥9.25% of immune infiltrate, n=41 tumors). Kaplan-Meier analysis demonstrated that increasing CD103 expression was significantly associated with reduced number of days to metastasis (p=0.0034, figure 6C). This association was more significant when the two groups were separated at the most informative cutpoint based on maximally selected rank statistics (p=0.0003, online supplemental figure 8A) and maintained significance when the cohort of cSCCs was split into three groups characterized by low (bottom 33%), medium (middle 33%) and high (top 34%) CD103+ cell frequencies (p=0.0003, online supplemental figure 8B). These results indicate that increased CD103 expression is associated with poorer clinical outcomes in cSCC.

10.1136/jitc-2020-001807.supp8 Supplementary data

To investigate the association between CD103 expression and survival in other cancer types, TCGA data were analyzed using TIMER2.0,^17^ demonstrating that high (greater than median) expression of *ITGAE* (encoding CD103) was associated with reduced survival in primary cutaneous melanoma, breast carcinoma, renal cell carcinoma, kidney chromophobe cancer and lower grade glioma, figure 6D, whereas two cancer types showed the opposite association (high *ITGAE* expression was associated with increased survival in cervical/endocervical cancer and pancreatic adenocarcinoma). Using a Cox proportional hazards model, higher *ITGAE* expression was also significantly associated with poorer survival in adrenocortical carcinoma as well as in primary cutaneous melanoma, breast carcinoma, kidney renal cell carcinoma and kidney chromophobe cancer (online supplemental figure 8C).

To determine whether the increased CD103 expression in P-M cSCCs was on CD8+ cell populations rather than on other cells, immunofluorescence microscopy was performed on P-NM (n=56) and P-M (n=47) cSCCs (figure 6E). Significantly higher frequencies of tumor-infiltrating CD8+ cells expressed CD103 in P-M than P-NM cSCCs (median 35.0% vs 20.4%, respectively, p<0.0001, figure 6F). There was no significant difference in CD8−CD103+ cell frequencies between P-M and P-NM cSCCs (2.0% vs 2.3% of immune infiltrate respectively, p=0.59, figure 6G). Increased CD8+CD103+ frequencies as a percentage of the CD8+ population was significantly associated with reduced time to metastasis (CD8+CD103+ high and CD8+CD103+ low representing above and below median value, respectively: p=0.0025, figure 6H). This difference in time to metastasis was more evident at the optimal cutpoint comparing the 20% of cSCCs with the highest CD8+CD103+ frequencies with the 80% of cSCCs with lower CD8+CD103+ frequencies (p<0.0001, figure 6I). When the cSCC cohort was split into three groups distinguished by CD8+CD103+ high (top 34%), medium (middle 33%) and low (bottom 33%) frequencies (p<0.0001), it suggested that most of the difference in time to metastasis was due to the CD8+CD103+ high group (online supplemental figure 8D). While the difference in 5-year overall survival between the cSCCs with CD8+CD103+ cell frequencies lower than median and those greater than median did not reach significance (p=0.0566, figure 6J), at the optimal cutpoint there was significantly reduced 5-year overall survival in the top 19% compared with the bottom 81% of cSCCs by CD8+CD103+ cell frequencies (p=0.0002, figure 6K). Similarly, although the 5-year disease-specific survival did not differ significantly between the CD8+CD103+ low and high groups when divided at the median value (p=0.0711. figure 6L), at the optimal cutpoint the top 27% of cSCCs according to CD8+CD103+ cell frequencies had significantly reduced 5-year disease-specific survival than the cSCCs with lower CD8+CD103+ cell frequencies (p=0.0022, figure 6M).

## Discussion

The role of immunosurveillance is essential in preventing cancer development, particularly in cSCCs, which are promoted greatly by immunosuppression. cSCCs are associated with an immune infiltrate which is ineffective at destroying the cancer, and we have shown previously that cSCCs are infiltrated with Langerhans cells and CD8+ T cells that protect against development of metastases and, conversely, immunosuppressive Tregs which express costimulatory receptors, such as OX40 and 4-1BB, and suppress antitumor immune responses, leading to metastasis.^16 20 21^ In addition, there are other immunopathogenic cells in cSCC that enable or promote tumor development, for example, γδ T cells, which may influence clinical outcome in cSCCs.^22 23^ It is also increasingly apparent that memory T cells play a key role in cancer immune surveillance,^8^ and PD-1 blockade for cancer immunotherapy activates and expands intratumoral memory T cells.^24^ An improved understanding of tumor-infiltrating memory T cells and characterization of the memory T-cell phenotypes, including those relevant to clinical outcome, is important for identification of potential immunotherapeutic targets in cancer.

Multiple functionally distinct memory T-cell subpopulations have been demonstrated previously in skin.^14^ Non-recirculating TRMs express CD69 and include CD103− TRM and CD103+ TRM subgroups, which have potent effector functions, whereas recirculating T cells in skin include TCMs and TMMs.^14^ In this study, we have performed in-depth characterization of the memory T-cell compartments within the CD4 and CD8 T-cell populations in cSCC. Our findings show that most cSCC T cells are of a CCR7−CC45RA− TEM phenotype, with smaller populations of CCR7+CD45RA+ naive T cells, CCR7−CD45RA+ TEMRAs, CCR7+CD45RA− TCMs and CCR7+L-selectin− TMMs present. Many tumor-infiltrating memory T cells expressed the TRM marker CD69, and TRMs were subcategorized as CD103+ or CD103−. CD8+CD103+ TRMs were increased in frequency in cSCCs compared with normal skin, and CD8+CD103+ TRMs demonstrated increased IL-10 production and expression of CD39, CTLA-4 and PD-1. As this suggests an immunosuppressive/inhibitory phenotype for CD8+CD103+ TRMs, we investigated their role in relation to clinical outcome in cSCC, and found that higher CD103 expression and higher CD103+ frequencies as a percentage of the CD8+ cell population were associated with the development of, and reduced time to, metastasis.

CD103, a well-characterized marker for TRMs, is an integrin that binds E-cadherin which enables TRMs to remain permanently in the peripheral tissues without recirculating.^25^ CD103 is required for TRM formation, which enables superior protection against cutaneous viral infections.^26^ CD103+ TRMs in skin are enriched in the epidermis and are associated with potent effector cytokine production.^14^ In our study, an increased frequency of CD103+CD8 T cells was identified in cSCC tumors compared with normal skin. Epidermal TGF-β has been shown to induce CD103 expression by skin T cells which enables tethering of CD103+ T cells within the epidermis,^14^ and it is known that cSCCs frequently overexpress TGF-β,^27^ which may lead to increased CD103 expression in cSCC. In addition, CD103+ tumor-infiltrating CD8+ T cells can self-regulate their CD103 expression by producing TGF-β.^28^ Furthermore, ultraviolet radiation-induced extracellular ATP release by keratinocytes activates skin resident T cells and upregulates CD69.^29^ Extracellular ATP also activates the purinergic receptor P2R×7, which is required for the generation and functionality of long-lived CD103+ TRMs in tissues.^30^

In the current study, we showed that CD8+CD103+ TRMs in cSCC were able to produce IFNγ, TNFα and IL-2, indicating that they may have some immunostimulatory abilities. Consistent with this, in lung cancer CD103+CD45RO+CD8+ T cells are the main source of IFNγ in tumor-infiltrating lymphocytes, and CD8+CD103+ T cells can augment cytotoxic CD8+ T-cell cancer recognition and enhance antitumor cytotoxicity.^28 31^ However, in the current study, we also noted that CD8+CD103+ TRMs in cSCC exhibited increased production of the immunosuppressive cytokine IL-10, the ectonucleotidase CD39 (a rate limiting enzyme for the generation of immunosuppressive adenosine) and upregulation of the exhaustion marker PD-1. Related to this, CD103+ TRM cells have been reported to have higher expression of inhibitory receptors such as CTLA4, Tim-3 and PD-1, as well as CD39.^28 31^ Likewise, CD103+CD39+ tumor-infiltrating CD8 T cells have been shown to be enriched for tumor-reactive cells and display exhausted gene signatures.^32^ This suggests that the role of CD103 on tumor-infiltrating T cells in cancer may be more complex than previously thought, and that tumor-reactive CD103+ TRMs may boost or inhibit the antitumor immune response.

Indeed, we showed that CD103+ TRMs are associated with poorer clinical outcomes in cSCC, which is in contrast to some studies on other types of cancer. It has been reported that CD8+CD103+ TRMs are critical for protection against melanoma in mice,^11 12^ and associated with improved survival in metastatic melanoma,^33^ although this may not be the case in primary cutaneous melanoma because TCGA data demonstrate reduced survival in primary cutaneous melanomas with high expression of *ITGAE*, which encodes for CD103 (figure 6H). CD103+ TRMs have also been described as protective or conveying better prognosis in other tumor types, including oropharyngeal,^34^ head and neck,^32^ lung,^35^ breast^36 37^ and ovarian cancers.^38^ However, by contrast, Gabriely *et al* showed that CD8+CD103+ T cells in murine melanomas had a regulatory phenotype which upregulated IL-10, CTLA4 and CD25, suppressed CD8+ T-cell proliferation and promoted tumor growth.^39^ This would be in keeping with the findings shown in our study, which suggest a regulatory phenotype for CD8+CD103+ TRMs in human cSCC. In addition, Gabriely and colleagues also demonstrated that high CD103 expression was associated with shorter survival in patients with glioma and glioblastoma.^39^ Consistent with this, we have shown that high CD103+/CD8+CD103+ expression is associated with the development of, and reduced time to, cSCC metastasis, suggesting that CD8+CD103+ TRMs comprise an important cell population involved in determining the development of metastasis in cSCC, and that these human CD8+CD103+ TRMs could be equivalent to the immunosuppressive murine CD8+CD103+ T-cell population identified by Gabriely *et al*.^39^ Alternatively, as CD39, CTLA-4 and PD-1 expression could denote recent activation of the cSCC CD8+CD103+ TRMs, the association between activated CD8+CD103+ TRMs with metastasis in cSCC might be explained by the increased selective pressure exerted by these TRMs on the tumor, causing loss of tumor antigens via immunoediting, driving tumor evolution to escape immune recognition,^40^ leading to subsequent development of metastasis. Further evidence for the association between CD103 and poorer clinical outcome in cancer was demonstrated in TCGA data showing high *ITGAE* expression was associated with reduced survival in primary cutaneous melanoma, breast carcinoma, kidney chromophobe cancer, renal cell carcinoma, lower grade glioma and adrenocortical carcinoma. Based on the associations of CD103+ TRMs with metastases and survival in different types of cancer, it remains unclear how CD103+ TRMs influences outcome in different cancers and whether the pathogenic role of CD103+ TRMs in diverse cancer types is tumor or organ-dependent, or differs between primary and metastatic tumors in some cancer types.

Our previous work showed that Tregs form a functionally important T-cell subgroup in cSCCs which suppress antitumor immunity and associate with development of metastasis.^16^ It has been shown that CD103 can be expressed by Tregs in murine cancers,^41^ but only 1.1% of tumor-infiltrating CD4+FOXP3+ Tregs were CD69+CD103+ in our study (figure 4C), indicating that the Tregs and CD103+ TRMs in cSCCs are separate cell populations. We have previously shown that cSCCs contain higher percentages of Tregs as a proportion of the CD4+ T-cell population than those in NS,^16^ and this increase in Tregs may explain the decreased CD4+CD69+CD103+ percentages in cSCC compared with NS in the current study (figure 3I). CD4+CD103+ TRMs in cSCC were able to produce IFNγ and TNFα (and also upregulated CD39 and PD-1, online supplemental figure 5), so it is possible that their reduction/suppression by Tregs is another mechanism for decreased immune surveillance in cSCC. Therefore, it is likely that a combination of defective immune responses, which include increased Tregs, reduced CD4+CD103+ TRMs and increased inhibitory CD8+CD103+ TRMs, provides an immunosuppressive environment in cSCC, which permits subsequent tumor progression and metastasis.

## Conclusions

We have performed in-depth characterization of the memory T-cell compartment in human cSCCs, highlighting CD8+CD103+ TRMs which express inhibitory receptors and suppressive markers as an important cell population which contributes to dysfunctional antitumor immunity. Furthermore, our results demonstrate that high CD8+CD103+ expression is associated with metastasis and poorer clinical outcome in this tumor. Our data when viewed in conjunction with previous studies on tumor-infiltrating CD8+CD103+ T cells suggest that, depending on the cancer type, CD8+CD103+ TRMs can either promote or inhibit the development of metastasis in cancer.
