# Supplementary material for: CD8+CD103+ tissue-resident memory T cells convey reduced protective immunity in cutaneous squamous cell carcinoma
Source: J Immunother Cancer. 2021 Jan 21;9(1):e001807. doi: 10.1136/jitc-2020-001807 (PMC7825273; doi:10.1136/jitc-2020-001807)
Supplement: Supplementary data [file jitc-2020-001807supp001.pdf]

Supplementary figure 1

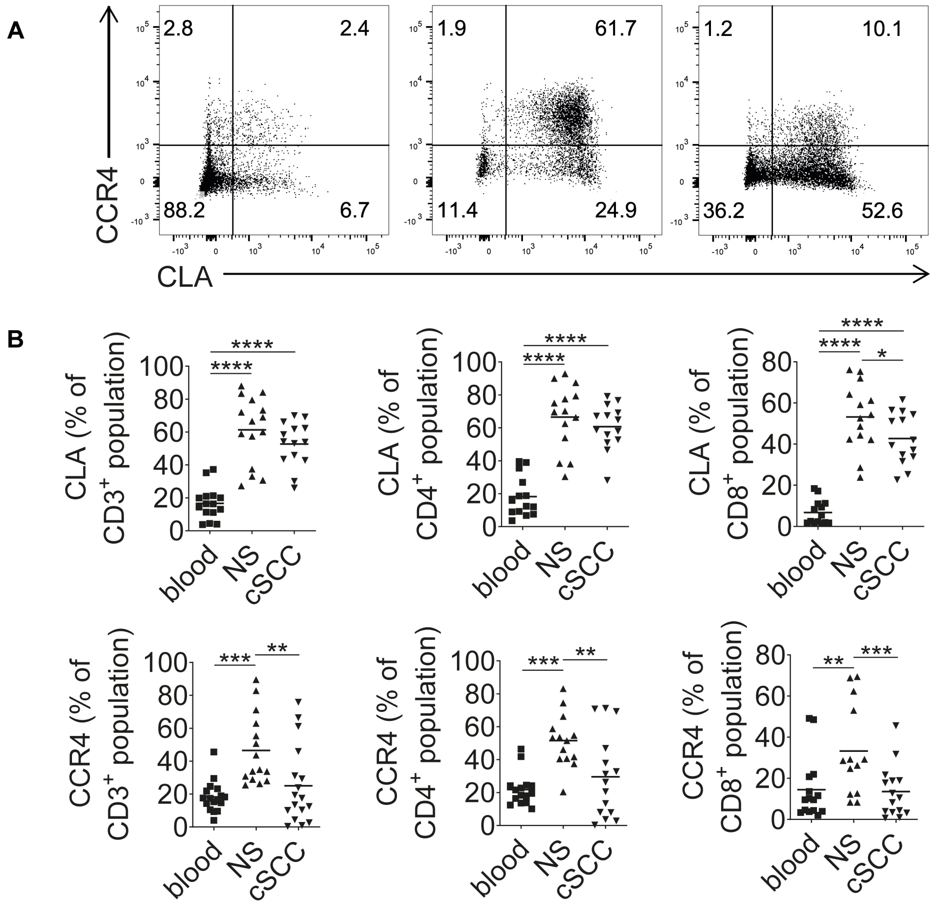

Supplementary Figure 1. Expression of CLA and CCR4 skin homing markers by T cells in blood, normal skin and cSCC. A) Representative FACS plots gated on CD3+ T cells from blood, normal skin (NS) and cSCC from the same patient showing expression of CLA (x axis) and CCR4 (y axis). (B) Graphs showing percentages of CD3+ (left), CD4+ (centre) and CD8+ (right) T cells from blood, normal skin (NS) and cSCC which express CLA (upper row, n=14 tumors) and CCR4 (lower row, n=17 tumors). Horizontal bars = means, *p<0.05, **p<0.01, ***p<0.001, ****p<0.0001.
