# Supplementary material for: CD8+CD103+ tissue-resident memory T cells convey reduced protective immunity in cutaneous squamous cell carcinoma
Source: J Immunother Cancer. 2021 Jan 21;9(1):e001807. doi: 10.1136/jitc-2020-001807 (PMC7825273; doi:10.1136/jitc-2020-001807)
Supplement: Supplementary data [file jitc-2020-001807supp002.pdf]

Supplementary figure 2

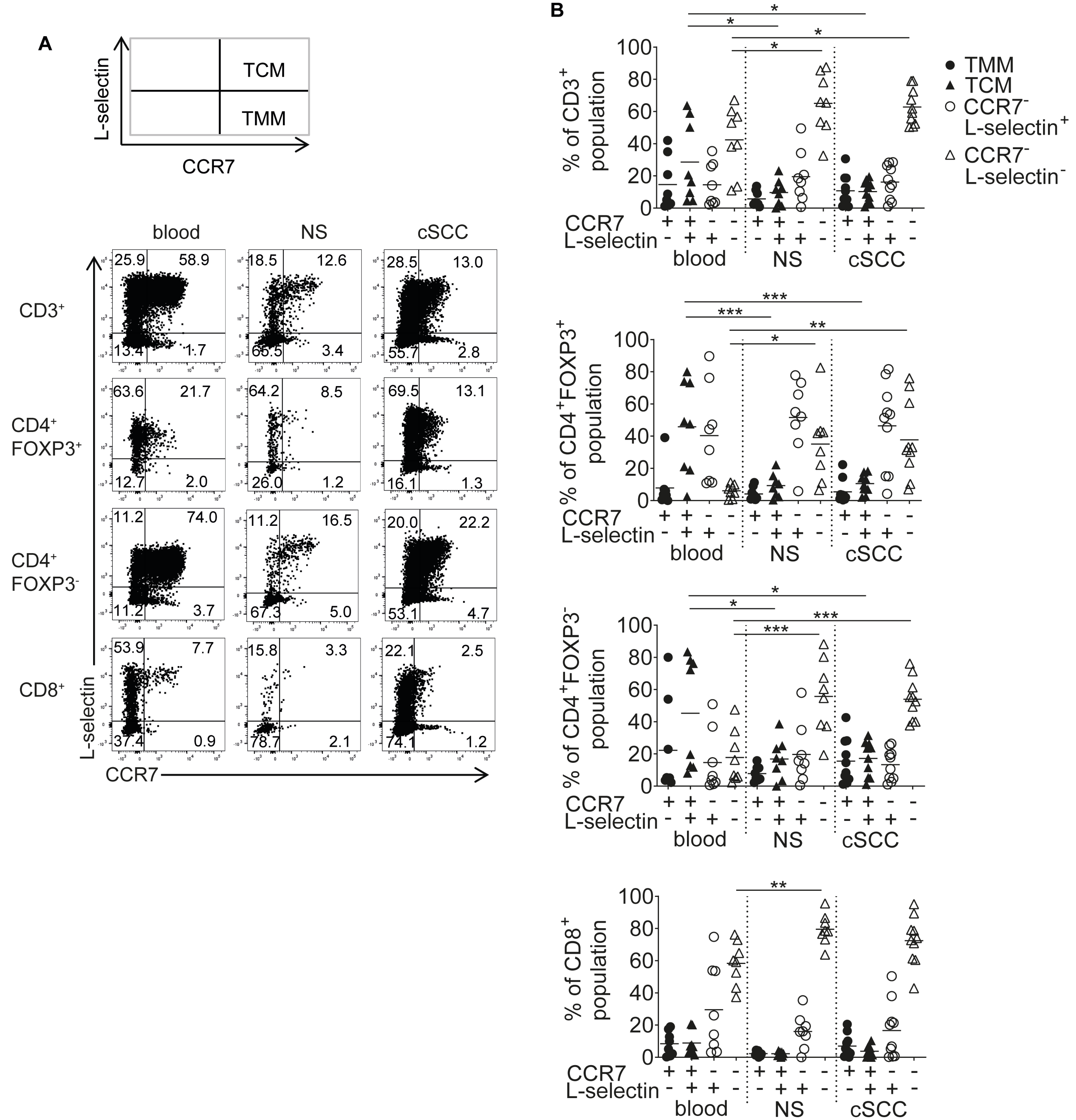

Supplementary Figure 2. CCR7 and L-selectin expression on T cells in blood, normal skin and cSCC. (A) Representative FACS plots from blood, normal skin (NS) and cSCC from the same patient showing expression of CCR7 (x axis) and L-selectin (y axis) in CD3+, CD4+FOXP3+, CD4+FOXP3- and CD8+ gated populations. A schematic diagram is shown above on top, defining the TCM and TMM populations by CCR7 and L-selectin expression as depicted. (B) Graphs showing percentages of CD3+, CD4+FOXP3+, CD4+FOXP3- and CD8+ T cells from blood, normal skin (NS) and cSCC (n=10 tumors) which are TMM (filled circle), TCM (filled triangle), CCR7-L-selectin+ (unfilled circle) and CCR7-L-selectin- (unfilled triangle). Horizontal bars = means, *p<0.05, **p<0.01, ***p<0.001.
