# Supplementary material for: CD8+CD103+ tissue-resident memory T cells convey reduced protective immunity in cutaneous squamous cell carcinoma
Source: J Immunother Cancer. 2021 Jan 21;9(1):e001807. doi: 10.1136/jitc-2020-001807 (PMC7825273; doi:10.1136/jitc-2020-001807)
Supplement: Supplementary data [file jitc-2020-001807supp003.pdf]

Supplementary figure 3

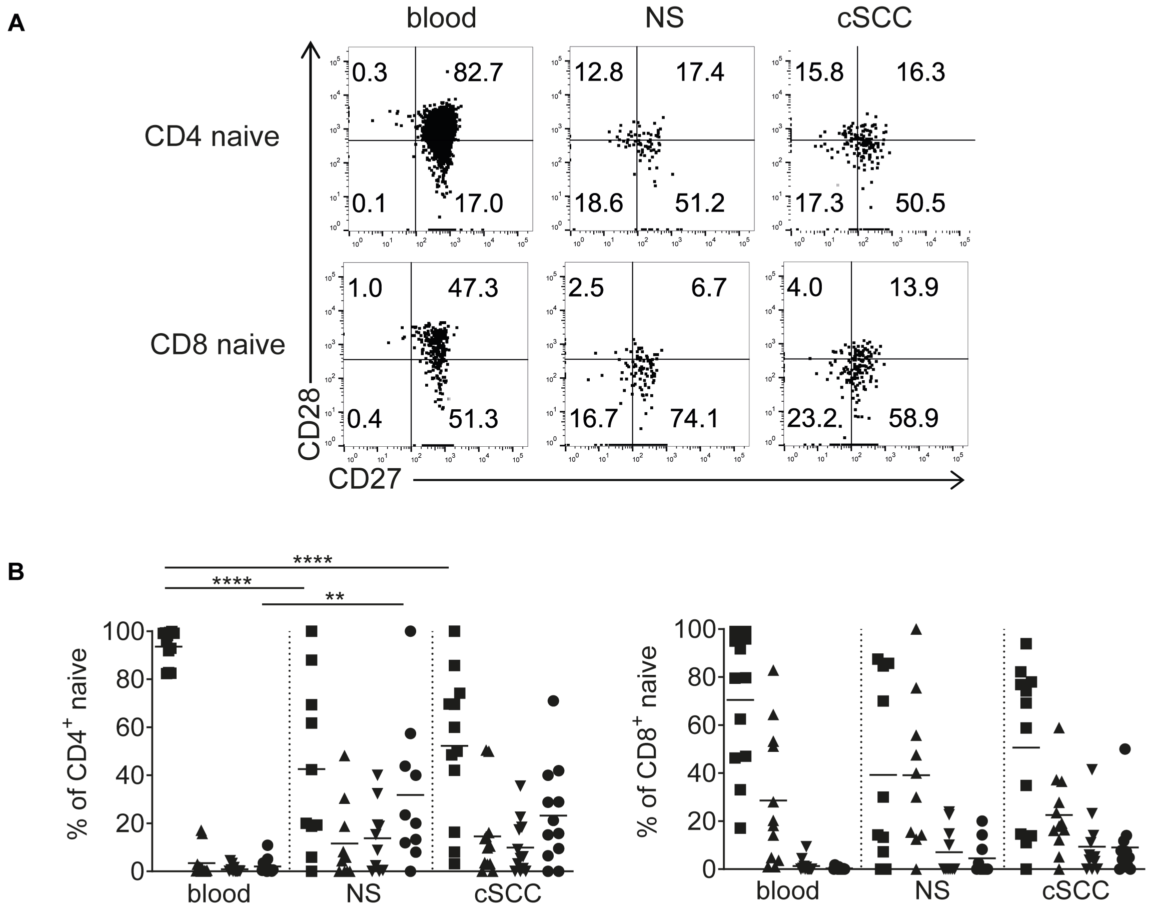

Supplementary Figure 3. Expression of CD27 and CD28 by naive T cells in blood, normal skin and cSCC. (A) Representative FACS plots of naive T cells from blood, normal skin (NS) and cSCC from the same patient showing expression of CD27 (x axis) and CD28 (y axis). (B) Graphs showing percentages of CD4+ naive T cells (left), and CD8+ naive T cells (right), from blood, normal skin (NS) and cSCC (n=14 tumors) which are CD27+CD28+, CD27+CD28-, CD27-CD28- and CD27-CD28+. Horizontal bars = means, **p<0.01, ****p < 0.0001.
