# Supplementary material for: CD8+CD103+ tissue-resident memory T cells convey reduced protective immunity in cutaneous squamous cell carcinoma
Source: J Immunother Cancer. 2021 Jan 21;9(1):e001807. doi: 10.1136/jitc-2020-001807 (PMC7825273; doi:10.1136/jitc-2020-001807)
Supplement: Supplementary data [file jitc-2020-001807supp004.pdf]

Supplementary figure 4

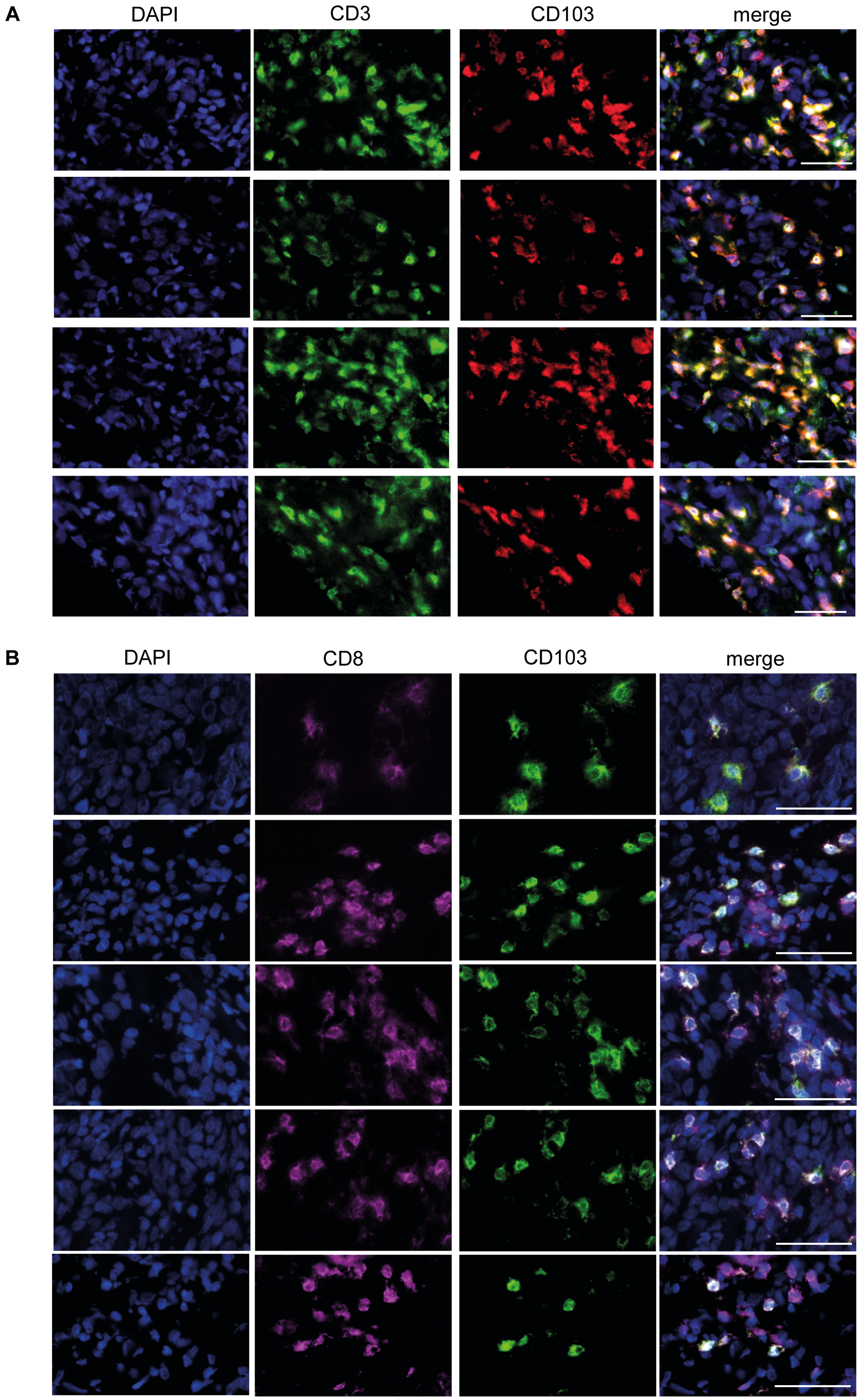

Supplementary Figure 4. Most CD103+ cells in cSCC are CD3+ and CD8+ T cells. Representative immunofluorescence microscopy images of cSCCs showing (A) CD3 and CD103 expression and (B) CD8 and CD103 expression. Scale bars = 50 µm.
