# Supplementary material for: CD8+CD103+ tissue-resident memory T cells convey reduced protective immunity in cutaneous squamous cell carcinoma
Source: J Immunother Cancer. 2021 Jan 21;9(1):e001807. doi: 10.1136/jitc-2020-001807 (PMC7825273; doi:10.1136/jitc-2020-001807)
Supplement: Supplementary data [file jitc-2020-001807supp005.pdf]

BMJ Publishing Group Limited (BMJ) disclaims all liability and responsibility arising from any reliance placed on this supplemental material which has been supplied by the author(s)

Supplementary figure 5

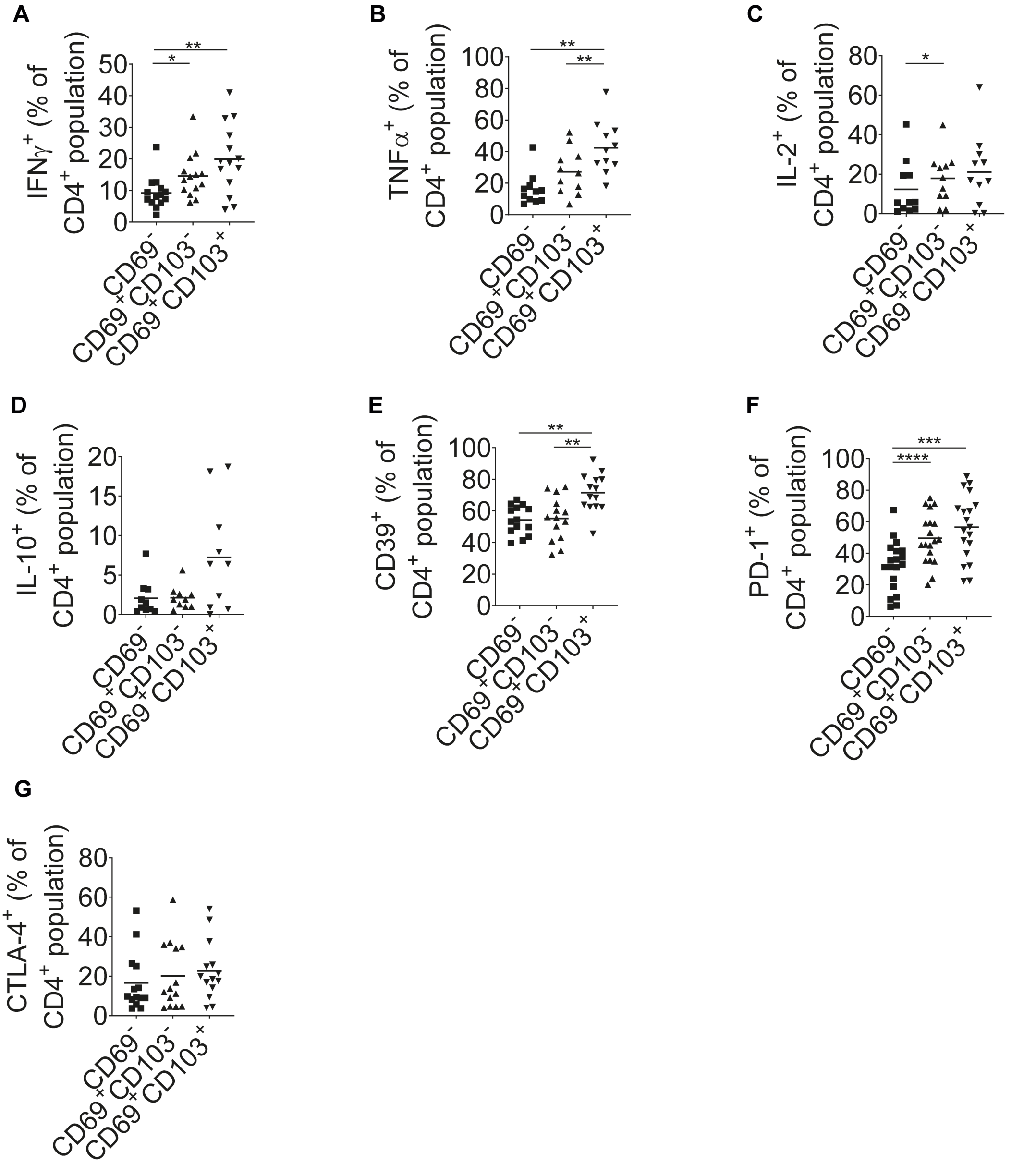

Supplementary Figure 5. Characterization of CD4+CD69+CD103+ T cells in cSCC. (A-G) Graphs showing expression of (A) IFNγ (n=14 tumors), (B) TNFα (n=11 tumors), (C) IL-2 (n=11 tumors), (D) IL-10 (n=10 tumors), (E) CD39 (n=14 tumors), (F) PD-1 (n=11 tumors) and (G) CTLA-4 (n=14 tumors) by the tumoral CD4+CD69-, CD4+CD69+CD103- and CD4+CD69+CD103+ T cell populations. Expression of cytokines was determined following stimulation in vitro with PMA and ionomycin for 5 hours. Horizontal bars = means, *p<0.05, **p<0.01, ***p<0.001, ****p<0.0001.
