# Supplementary material for: CD8+CD103+ tissue-resident memory T cells convey reduced protective immunity in cutaneous squamous cell carcinoma
Source: J Immunother Cancer. 2021 Jan 21;9(1):e001807. doi: 10.1136/jitc-2020-001807 (PMC7825273; doi:10.1136/jitc-2020-001807)
Supplement: Supplementary data [file jitc-2020-001807supp006.pdf]

Supplementary figure 6

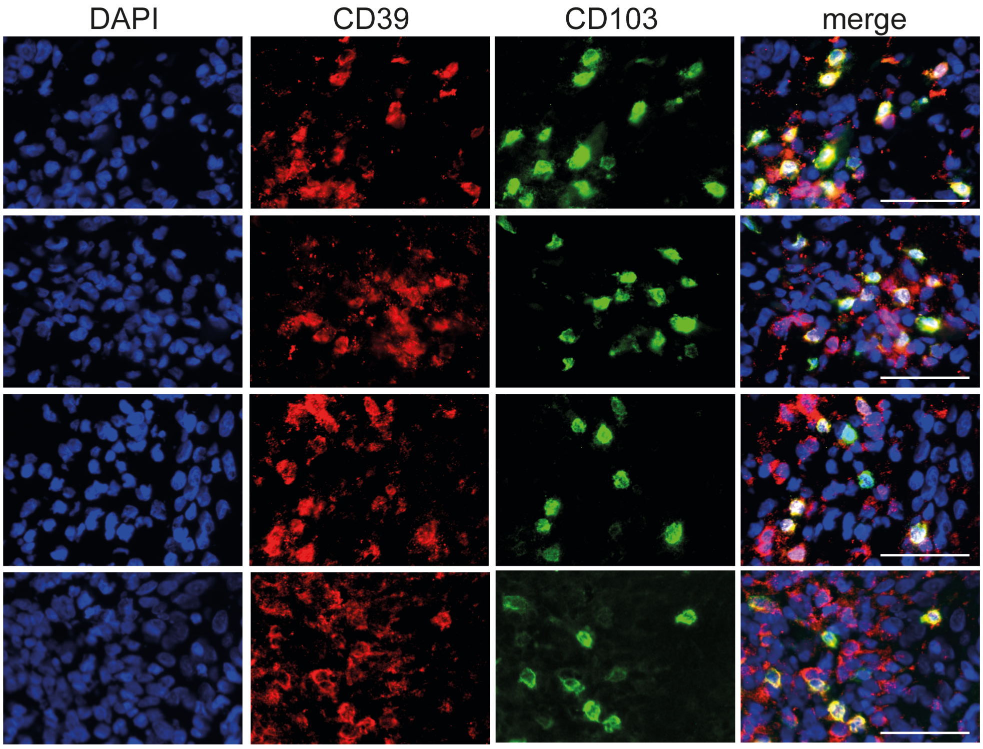

Supplementary Figure 6. Expression of CD39 by CD103+ T cells in cSCC. Representative immunofluorescence microscopy images of cSCC stained for CD103 and CD39. Scale bars = 50 μm.
