# Supplementary material for: CD8+CD103+ tissue-resident memory T cells convey reduced protective immunity in cutaneous squamous cell carcinoma
Source: J Immunother Cancer. 2021 Jan 21;9(1):e001807. doi: 10.1136/jitc-2020-001807 (PMC7825273; doi:10.1136/jitc-2020-001807)
Supplement: Supplementary data [file jitc-2020-001807supp007.pdf]

Supplementary figure 7

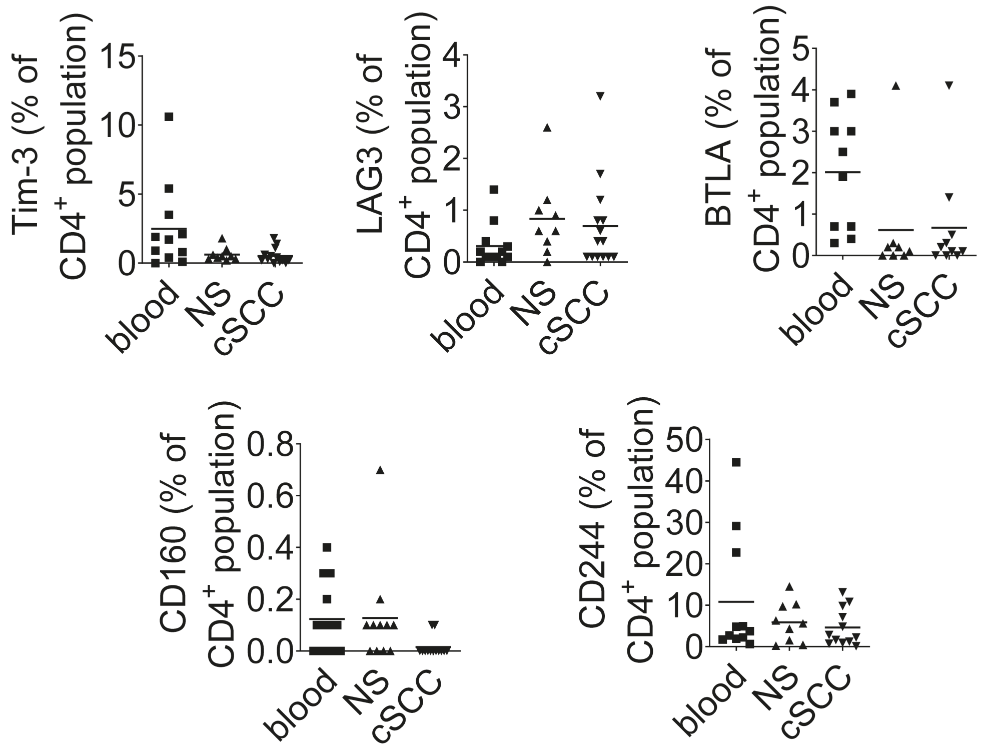

Supplementary Figure 7. Expression of inhibitory receptors by CD4+ T cells in cSCC. Percentages of CD4+ T cells from blood, normal skin (NS) and cSCC expressing Tim3 (upper left, n=13 tumors), LAG3 (upper centre, n=14 tumors), BTLA (upper right, n=11 tumors), CD160 (lower left, n=13 tumors) and CD244 (lower right, n=12 tumors). Horizontal bars = means.
