# Supplementary material for: CD8+CD103+ tissue-resident memory T cells convey reduced protective immunity in cutaneous squamous cell carcinoma
Source: J Immunother Cancer. 2021 Jan 21;9(1):e001807. doi: 10.1136/jitc-2020-001807 (PMC7825273; doi:10.1136/jitc-2020-001807)

Supplementary figure 8

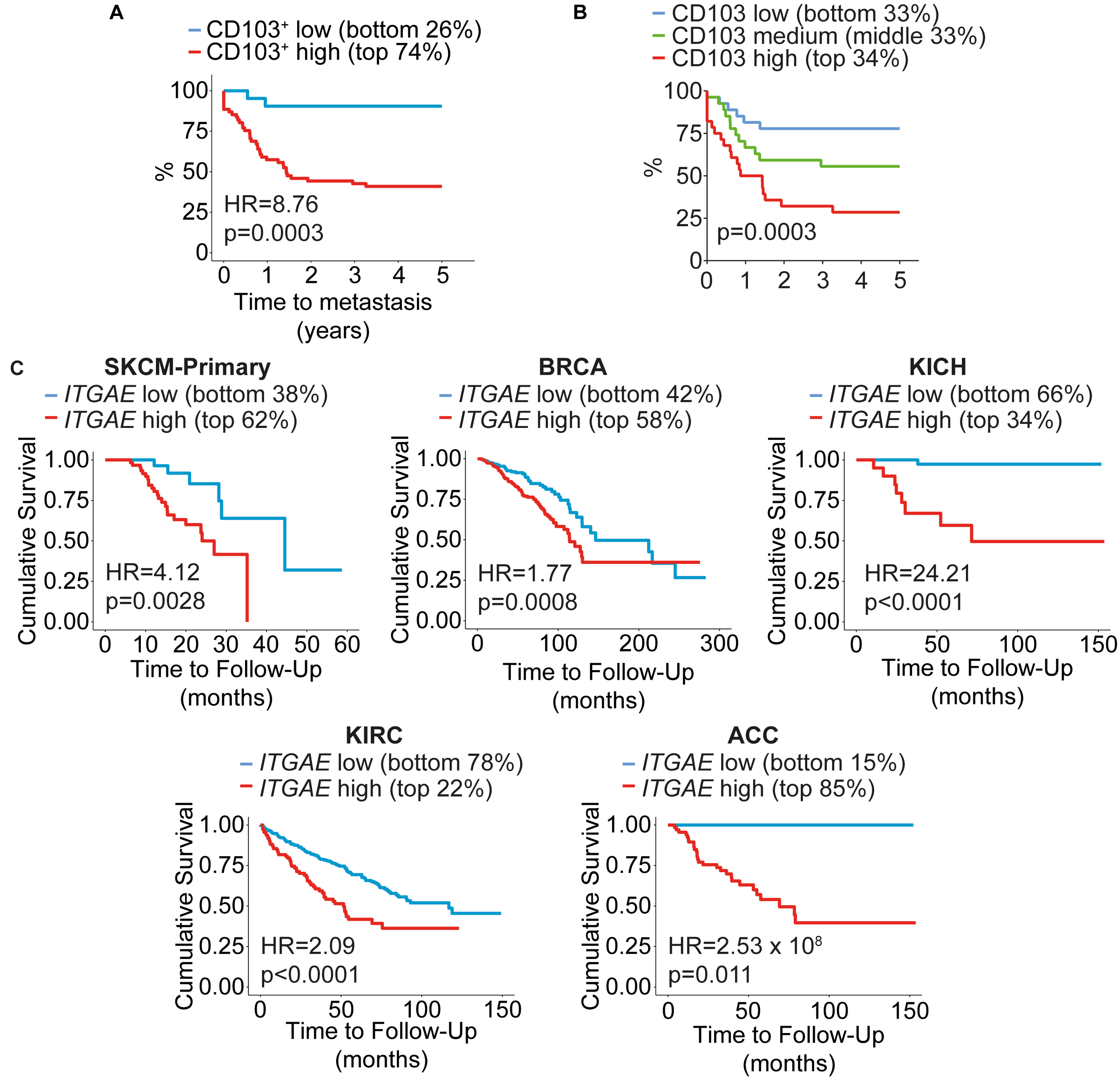

Supplementary Figure 8. CD103 expression associates with poorer clinical outcome in cSCC and other cancer types. (A, B) Time to metastasis for cSCCs in figure 6B split into (A) low and high CD103+ cell frequencies divided at the most informative cutpoint based on maximally selected rank statistics (low <5.8% of immune infiltrate, n=21; high ≥5.8% of immune infiltrate, n=61) and (B) stratified by low (<6.5% of immune infiltrate, n=27), medium (6.5 – 11.7% of immune infiltrate, n=27) and high (>11.7% of immune infiltrate, n=28) expression of CD103. (C) TCGA data showing cumulative survival based on high and low expression of ITGAE split at the most informative cutpoint determined by maximally selected rank statistics (SKCM-Primary = primary skin melanoma; BRCA = breast carcinoma; KICH = kidney chromophobe cancer; KIRC = kidney renal clear cell carcinoma; ACC = adrenocortical carcinoma). (D) Time to metastasis for cSCCs in figure 6F stratified by low (<19.0% of CD8+ population, n=34), medium (19.0 – 34.7% of CD8+ population, n=34) and high (>34.7% of CD8+ population, n=35).

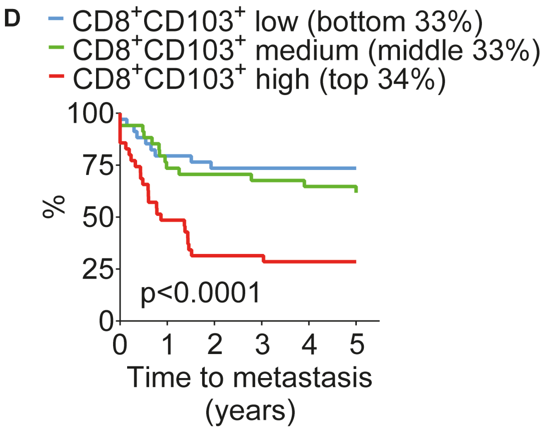

Supplement: Supplementary data [file jitc-2020-001807supp008.pdf]
